# Closed-loop synchronization versus conventional synchronization in spontaneously breathing pediatric patients (CHESTSIPP) – a randomized controlled cross-over study

**DOI:** 10.3389/fmed.2026.1745939

**Published:** 2026-03-25

**Authors:** Gokhan Ceylan, Ozlem Sarac, Gulhan Atakul, Ekin Soydan, Mustafa Colak, Sevgi Topal, Pinar Hepduman, Dominik Novotni, Johannes Meyer, Utku Karaaslan, Jean-Pierre Revely, Hasan Ağın

**Affiliations:** 1 Department of Paediatric Intensive Care Unit, Dr. Behçet Uz Children’s Diseases and Surgery Training and Research Hospital, University of Health Sciences, Izmir, Türkiye; 2 Department of Medical Research, Hamilton Medical AG, Bonaduz, Switzerland; 3 Department of Paediatric Intensive Care Unit, Cam Sakura Training and Research Hospital, University of Health Sciences, Istanbul, Türkiye; 4 Department of Paediatric Intensive Care Unit, Acibadem Altunizade Hospital, Acibadem University, Istanbul, Türkiye; 5 Department of Paediatric Intensive Care Unit, Erzurum Territorial Training and Research Hospital, University of Health Sciences, Erzurum, Türkiye

**Keywords:** asynchronies, closed-loop, mechanical ventilation, pediatric acute respiratory distress syndrome (PARDS), spontaneous breathing, synchronization, waveform analysis

## Abstract

**Background:**

Patient–ventilator asynchrony (PVA) is common during invasive mechanical ventilation in children and may increase respiratory workload and discomfort. The IntelliSync+ (IS+) algorithm provides closed-loop synchronization by continuously analyzing airway pressure and flow waveforms to optimize inspiratory and expiratory cycling in real time. This study evaluated the efficacy and safety of IS+ compared with conventional physician-tailored synchronization in spontaneously breathing pediatric patients.

**Methods:**

Multicenter, prospective, randomized controlled, single-blind crossover trial conducted in four pediatric intensive care units between March 2024 and May 2025. Patients aged 1 month to 18 years undergoing invasive mechanical ventilation were randomized to begin with either IS+ or conventional synchronization. Each mode was applied for 90 min (30-min run-in and 60-min measurement), separated by a 30-min washout period. The primary outcome was the Asynchrony Index (AI). Secondary outcomes included major and minor asynchrony indices, Comfort-B scores, end-tidal carbon dioxide (EtCO_2_), oxygen saturation (SpO_2_), and leak percentage.

**Findings:**

Twenty-five patients completed both study phases. Compared with conventional synchronization, IS+ significantly reduced the overall AI (median 5.1% [IQR 4.2–6.4] vs. 12.4% [IQR 9.9–15.6]; *P* < 0.001), as well as both major and minor asynchrony indices (*P* < 0.001 for both). The frequencies of double triggering, auto-triggering, trigger delay, and early cycling decreased significantly, while ineffective efforts were unchanged and late cycling slightly increased. Comfort-B scores improved modestly (*P* = 0.02) and EtCO_2_ decreased (*P* < 0.001), whereas SpO_2_ and leak remained stable.

**Conclusion:**

Closed-loop synchronization with IS+ significantly improved patient–ventilator interaction without compromising comfort, oxygenation, or safety. These results support closed-loop synchronization as a feasible and physiologically sound strategy for improving ventilatory support quality in pediatric invasive mechanical ventilation.

**Clinical trial registration:**

[ClinicalTrials.gov], identifier [NCT05731024].

## 1 Introduction

Patient–ventilator asynchrony (PVA) is a frequent and clinically important problem in pediatric mechanical ventilation. Children have unique physiological characteristics, including high respiratory rates, small tidal volumes, and potential airway leaks from uncuffed tubes, which predispose them to mismatches between ventilator-delivered breaths and patients’ spontaneous efforts (1, 2). In pediatric cohorts, studies have demonstrated that approximately one in three ventilator breaths are asynchronous, underscoring the magnitude of the problem in intensive care settings (3). Conventional ventilator waveform monitoring alone is often insufficient to reliably detect all types of asynchronies in children (2, 4).

Addressing PVA is crucial, as it has been associated with prolonged duration of mechanical ventilation, higher ICU mortality, and increased hospital mortality (5, 6). Recent advancements in ventilator technology and clinical protocols have aimed to mitigate PVA by optimizing ventilator settings and improving patient–ventilator interaction. However, the effectiveness of these interventions in reducing PVA and improving outcomes remains an area of active investigation.

The IntelliSync+ (IS+) algorithm was based on real-time waveform analysis and developed to enhance patient–ventilator synchrony by continuously analyzing flow and pressure waveforms more than one hundred times per second. It automatically detects the onset of patient effort and initiates inspiration and expiration in real time, replacing fixed conventional trigger thresholds. The algorithm can be activated for the inspiratory trigger, expiratory trigger, or both (7, 8).

Recent studies in invasively ventilated patients have shown that IS+ improves triggering compared with conventional pressure-support ventilation (9). In bench studies simulating noninvasive ventilation (NIV) conditions, IS+ improved both inspiratory and expiratory synchrony across normal, obstructive, and restrictive respiratory profiles and maintained performance even with substantial leaks up to 20 L/min (10, 11).

In pediatric intensive care, patient–ventilator interaction during assisted ventilation is often suboptimal (12). Observational studies have reported high frequencies of depressed ventilatory drive and significant proportions of ventilation time spent in asynchrony under standard control modes (12). A multicenter pediatric trend analysis identified a 33% rate of asynchronous breaths and found that asynchrony prevalence increased over time, suggesting a cumulative effect or progressive mismatch (3).

There has been a persistent gap in research evaluating the effectiveness and safety of closed-loop synchronization systems in pediatric patients receiving invasive mechanical ventilation, particularly in those with spontaneous respiratory effort. While adaptive and closed-loop ventilator strategies have been investigated in adult populations, evidence in children remains limited, and systematic reviews have suggested potential benefits of automation in reducing ventilation duration and associated complications (13).

To address this knowledge gap, we conducted a prospective, randomized controlled cross-over study designed to compare the performance of a closed-loop synchronization system (IS+) with conventional clinician-controlled synchronization during invasive mechanical ventilation in spontaneously breathing pediatric patients admitted to the pediatric intensive care unit (PICU). Each patient underwent both ventilation strategies in a randomized order, enabling within-subject comparison of synchronization quality, physiological stability, and safety.

The primary focus of the study was to determine whether IS+ could reduce patient–ventilator asynchrony compared with conventional synchronization control. Secondary analyses explored parameters related to patient comfort, ventilatory performance, and gas-exchange efficiency. We hypothesized that activation of IS+ would improve patient–ventilator synchrony without compromising safety in this vulnerable population.

## 2 Methods

### 2.1 Study design

This study adopted a multicenter, single-blinded, randomized, cross-over design to compare closed-loop synchronization using IS+ with conventional clinician-controlled synchronization in the pediatric population undergoing invasive mechanical ventilation. The trial was conducted across four tertiary pediatric intensive care units (PICUs) in Turkey: Dr. Behçet Uz Children’s Research and Training Hospital in Ýzmir, Aydın Obstetrics and Children Hospital in Aydın, Erzurum Territorial Training and Research Hospital in Erzurum, and Başakşehir Çam and Sakura Research and Training Hospital in Istanbul.

The enrolment period extended from April 2023 to May 2025. Ethical approval for the study was obtained from the Institutional Ethics Committee of Dr. Behçet Uz Children’s Hospital (Approval No: 02019336, Board Affiliation: Ýzmir Health Sciences University). The study was conducted in accordance with the principles outlined in the Declaration of Helsinki. The trial was prospectively registered on ClinicalTrials.gov under the identifier NCT05731024.

### 2.2 Participants

Patients were eligible for inclusion if they were older than 1 month and younger than 18 years of age, hospitalized in the pediatric intensive care unit (PICU) with an anticipated requirement for invasive mechanical ventilation for at least the following 3 h while exhibiting spontaneous breathing activity, and had written informed consent obtained and dated by the patient or by a legal guardian in cases where the patient was unable to provide consent. Before enrollment, all participants or their guardians received a complete verbal and written explanation of the study by the investigator.

Patients were excluded if there was a formalized ethical decision to withhold or withdraw life support, concurrent participation in another interventional research study, or previous enrollment in the present study during an earlier episode of respiratory failure. Additional exclusion criteria included pregnancy, an anticipated need for transfer from the PICU to another unit or hospital during the study period, or hemodynamic instability defined as a continuous infusion of epinephrine or norepinephrine infusion >2 μg/kg/m. Patients were also excluded if a reliable reference waveform from the esophageal pressure (Pes) signal could not be obtained, if the asynchrony index during baseline measurements was below 10%, if they had received any neuromuscular blocking agent within the last 12 h before study initiation, or if they had a known neuromuscular disorder affecting the diaphragm or other respiratory muscles.

These inclusion and exclusion criteria were established to ensure the validity of the synchronization assessment, maintain consistency in spontaneous respiratory effort across study participants, and uphold patient safety throughout the study.

### 2.3 Intervention

All patients were ventilated using invasive mechanical ventilation in pressure support mode. The applied pressure support (PS, cmH_2_O), positive end-expiratory pressure (PEEP, cmH2O), fraction of inspired oxygen (FiO_2_), and pressure rise time (P-ramp, ms) were determined by the attending physician according to clinical needs and were kept constant throughout the study. During both intervention periods, settings related to flow trigger (FT, L/min) and expiratory trigger sensitivity (ETS, %) differed according to the synchronization mode applied.

In the conventional synchronization period, FT and ETS adjustments were made manually by the attending physician based on the patient’s clinical condition. In contrast, during the closed-loop synchronization period, triggering and cycling were automatically managed by the IS+ algorithm, which continuously analyzed airway pressure and flow waveforms to detect patient efforts and to initiate and terminate inspiration and expiration in real time.

Before the intervention periods, a 30-min baseline measurement phase was performed under conventional ventilation using each patient’s routine ventilator settings as applied prior to study enrollment. This phase aimed to record reference asynchrony data and to confirm patient stability before initiating the randomized interventions. Following the baseline assessment, a 30-min run-in period was conducted under the first assigned synchronization mode according to randomization, allowing physiological adaptation and stabilization before formal data collection. Each intervention phase therefore lasted a total of 90 min, comprising a 30-min adaptation (run-in) phase followed by a 60-min measurement period used for comparative analysis. The 30-min washout interval between the two interventions simultaneously served as the run-in period for the subsequent phase, ensuring re-stabilization before the next measurement. After completion of both intervention phases and the final recordings, the study protocol concluded, and patients continued with conventional invasive mechanical ventilation and standard clinical management as prescribed by the treating team (Supplementary Figure 1).

### 2.4 Randomization and blinding

Participants who were already intubated and receiving invasive mechanical ventilation (IMV) as part of their routine clinical management were randomly assigned to begin with either a 1-h session of closed-loop synchronization using IS+ or a 1-h session of conventional synchronization. After completion of the first intervention, patients were transitioned to the alternative synchronization mode following a 30-min washout period. Randomization was performed in a 1:1 ratio using blocks of four and sealed opaque envelopes prepared by an independent investigator who was not involved in patient care or outcome assessment.

Due to the nature of the intervention, complete blinding of healthcare professionals was not feasible, as the synchronization mode required direct ventilator adjustments. However, patients were unaware of which synchronization mode was active during each intervention period. Furthermore, all investigators responsible for data collection and analysis were blinded to the intervention sequence to minimize observer bias.

### 2.5 Procedures

All patients were already intubated with an appropriately sized endotracheal tube before inclusion in the study. Throughout the trial, patients were maintained in a semi-recumbent position. Invasive mechanical ventilation was performed using a pediatric ventilator (Hamilton-C6, Hamilton Medical AG, Bonaduz, Switzerland) equipped with the IS+ closed-loop synchronization algorithm. Sedation and analgesia were administered as clinically indicated to ensure patient comfort and tolerance of invasive ventilation. The depth and regimen of sedation were maintained unchanged from baseline throughout the entire study period to avoid potential confounding effects on respiratory drive or synchrony.

An esophageal balloon catheter was inserted in all participants for measurement of esophageal pressure (Pes). Depending on patient size, either a seven French SmartCath pediatric catheter with a gastric feeding lumen (Vyaire Medical, Mettawa, IL, United States) or a five French adult catheter (Cooper-Surgical, Trumbull, CT, United States) was used, the latter typically in larger pediatric patients. Proper catheter positioning and balloon filling volume were determined using a modified Mojoli method, which followed a decremental approach in which the balloon was initially overinflated and then gradually deflated in a stepwise manner to identify the volume range providing optimal Pes signal amplitude and waveform morphology. This method ensured accurate signal acquisition for assessment of patient–ventilator interaction (14, 15).

The endotracheal tube and cuff pressure were adjusted to maintain air leaks below 20% during both intervention phases. A continuous cuff pressure control system (IntelliCuff, Hamilton Medical AG, Bonaduz, Switzerland) integrated to ventilator was used to maintain stable cuff pressurization and minimize leak variability throughout the study.

Routine patient care, including suctioning of airway secretions, administration of enteral or parenteral nutrition, and other nursing interventions, was performed as needed and distributed randomly across both intervention periods to avoid systematic bias. Periods during which esophageal pressure or ventilator waveforms were not clearly recordable because of routine patient care activities or artifacts were excluded from subsequent analysis. Staffing ratios remained consistent throughout the study: approximately six patients per physician and two patients per nurse during daytime shifts, and twelve patients per physician and three patients per nurse during night shifts. No additional research personnel were present during either intervention phase, ensuring that patient management reflected standard clinical practice.

### 2.6 Data collection

Case report forms (CRFs) were used to record clinical and demographic data for all participants. Ventilatory and physiological signals were continuously recorded via a Memory Box (Hamilton Medical AG, Bonaduz, Switzerland) connected to the ventilator’s RS-232 interface. The Memory Box operated in mixed mode, providing breath-by-breath monitoring of ventilatory values and high-frequency acquisition (50 Hz) of airway pressure and flow signals. This setup allowed precise temporal alignment of all waveform data and enabled detailed analysis of patient-ventilator interaction.

Esophageal pressure (Pes) signals obtained from the balloon catheter were integrated directly into the ventilator’s monitoring system and captured simultaneously through the same recording interface, ensuring complete synchronization between Pes and ventilator waveforms.

At the end of each intervention period, patient comfort was assessed using the Comfort-B (Behavioral) Scale, a validated tool for evaluating sedation and distress levels in pediatric intensive care patients.

### 2.7 Outcomes

The primary outcome was the Asynchrony Index (AI), defined as the proportion of asynchronous breaths, including both major and minor asynchronies, relative to the total number of recorded breaths plus ineffective efforts (16). It was calculated using the following formula:


$$A\,I=\left(\frac{m\,a\,j\,o\,r\,a\,s\,y\,n\,c\,h\,r\,o\,n\,y\,e\,v\,e\,n\,t\,s+m\,i\,n\,o\,r\,a\,s\,y\,n\,c\,h\,r\,o\,n\,y\,e\,v\,e\,n\,t\,s}{n\,u\,m\,b\,e\,r\,o\,f\,m\,a\,c\,h\,i\,n\,e\,b\,r\,e\,a\,t\,h\,s+i\,n\,e\,f\,f\,e\,c\,t\,i\,v\,e\,e\,f\,f\,o\,r\,t\,s}\right)\times 100$$

Secondary outcomes included several parameters. The Major Asynchrony Index was calculated as the percentage of major asynchrony events—specifically auto-triggering (AT), double breath (DB), and ineffective efforts (IE)—relative to the total number of breaths plus ineffective efforts:


$$M\,a\,j\,o\,r\,A\,I=\left(\frac{m\,a\,j\,o\,r\,a\,s\,y\,n\,c\,h\,r\,o\,n\,y\,e\,v\,e\,n\,t\,s}{n\,u\,m\,b\,e\,r\,o\,f\,m\,a\,c\,h\,i\,n\,e\,b\,r\,e\,a\,t\,h\,s+i\,n\,e\,f\,f\,e\,c\,t\,i\,v\,e\,e\,f\,f\,o\,r\,t\,s}\right)\times 100$$

The Minor Asynchrony Index was calculated for minor asynchronies, including early cycling (EC), late cycling (LC), and trigger delay (TD):


$$M\,i\,n\,o\,r\,A\,I=\left(\frac{m\,i\,n\,o\,r\,a\,s\,y\,n\,c\,h\,r\,o\,n\,y\,e\,v\,e\,n\,t\,s}{n\,u\,m\,b\,e\,r\,o\,f\,m\,a\,c\,h\,i\,n\,e\,b\,r\,e\,a\,t\,h\,s+i\,n\,e\,f\,f\,e\,c\,t\,i\,v\,e\,e\,f\,f\,o\,r\,t\,s}\right)\times 100$$

The Comfort-B (Behavioral) Scale, used to evaluate sedation depth and patient comfort at the end of each intervention period. Scores below 10 indicate possible over-sedation, scores between 12 and 17 indicate adequate comfort, and scores above 17 suggest agitation or inadequate sedation.

The percentage of leak around the endotracheal tube (%), as automatically calculated by the ventilator software on a breath-by-breath basis.

The mean peripheral oxygen saturation (SpO_2_, %), continuously monitored by the ventilator’s integrated pulse oximetry module and averaged over each 1-h period.

The mean end-tidal carbon dioxide (EtCO_2_, mmHg), recorded continuously through the ventilator’s mainstream capnography system and averaged across each 1-h intervention phase.

All outcomes were assessed over 1-h intervention periods for both synchronization modes.

### 2.8 Labeling of asynchronies

We applied the waveform analysis method described by Mojoli et al. to identify and classify asynchrony events (8). In addition to the ventilator-derived flow and airway pressure signals, the esophageal pressure (Pes) signal was used as a reference to verify patient effort and confirm the accuracy of waveform interpretation. This ensured physiologic validation of all detected events and increased the reliability of asynchrony classification.

The waveform analysis method was applied directly to invasive ventilation in this study to assess breathing patterns and ventilator synchronization. It relies on five fundamental principles: (I) normal breathing consists of active inspiration followed by passive expiration, (II) exponential decay of flow over time indicates passive conditions, (III) during well-synchronized pressure support ventilation, passive conditions are absent in the inspiratory phase but present in the expiratory phase, (IV) the presence of passive conditions during inspiration suggests auto-triggering (AT) or late cycling (LC), and (V) deviations from passive conditions during expiration indicate early cycling (EC) or ineffective efforts (IE) (8).

The onset of the patient’s inspiration was identified by a positive flow deflection and/or a negative deflection in airway pressure, while the end of inspiration was marked by the onset of exponential flow decay. Under optimal cycling conditions, exponential decay follows immediately after the peak expiratory flow. Termination errors, including early cycling (EC) and late cycling (LC), as well as ineffective efforts (IE), produce distinct deviations from this expected pattern.

Ineffective efforts were identified using two complementary criteria. In the absence of esophageal pressure monitoring, IE was recognized as a deviation from the expected expiratory flow pattern, characterized by a notch or bump in expiratory flow toward zero combined with an abrupt drop in airway pressure of at least 0.5 cmH2O (17, 18). When esophageal pressure (Pes) was available, detection was based on direct observation of inspiratory efforts in the Pes waveform that were not followed by a ventilator-triggered inspiration within a defined delay after the onset of effort. Each IE was counted as one asynchrony event per breathing cycle. During the expiratory phase, artifacts caused by cough or active expiratory efforts were excluded from analysis.

Trigger timing was evaluated according to ventilator response time. A response time between 0 and 117 ms was considered normal, between 118 and 234 ms was defined as trigger delay (TD), and greater than 234 ms was categorized as an ineffective effort (IE) (3, 12, 19). Early trigger events were labeled as auto-triggering (AT), characterized by an increase in flow, volume, and pressure without any corresponding patient effort. AT events were identified by the absence of a negative deflection in both airway pressure and esophageal pressure (Pes) and, in some cases, by the presence of a positive Pes deflection, indicating no inspiratory muscle activity. These events occurred asynchronously with the ventilator’s preset frequency.

Double breath (DB) was defined as two consecutive ventilator cycles separated by a brief expiratory time equal to or less than half of the patient’s inspiratory time (Ti) (17). The first breath had to be either patient- or ventilator-triggered; however, if the second cycle resulted from a patient effort occurring after an auto-trigger or mandatory breath, the event was still categorized as double triggering (20).

Late cycling (LC) was defined as a patient expiratory effort beginning before the ventilator’s inspiratory phase had terminated, typically manifested as a spike in airway pressure and a rapid decline in inspiratory flow near the end of inspiration (21). Early cycling (EC) was identified according to several waveform and physiologic features. On the flow–time curve, EC appeared as a sharp decrease from the peak expiratory flow lasting a few milliseconds, followed by an increase and then a gradual decline toward zero, indicating residual inspiratory muscle activity after the opening of the exhalation valve (22). It was also recognized by an upward concavity on the pressure waveform and a downward concavity on the flow waveform at the initiation of expiration (23), or by an upward “bump” on the expiratory flow portion of the flow–time curve (24). When Pes monitoring was available, EC was quantitatively defined by a delay between the end of patient inspiration and the end of ventilator inspiration: if ventilator cycling preceded patient inspiratory termination by ≥100 ms, early cycling was diagnosed. The end of patient inspiration was identified on the Pes signal as the midpoint of the rapid pressure rise following its inspiratory nadir (25).

All waveform classifications were verified using simultaneous esophageal pressure (Pes) recordings to ensure accurate identification and consistent interpretation of patient–ventilator asynchrony.

### 2.9 Power calculation

The determination of the sample size was based on data recorded from a pilot study involving seven patients (7 × 2 = 14 h), corresponding to over 24,000 analyzed breaths. Each participant completed two consecutive 1-h sessions comparing closed-loop synchronization using IS+ and conventional synchronization control. The pilot analysis aimed to evaluate the difference in the Asynchrony Index (AI) between the two synchronization modes. Utilizing the data from this pilot phase, a G*Power analysis was performed for a Wilcoxon signed-rank test (two-tailed, α = 0.05). The analysis indicated that 21 analyzable patient pairs would be required to achieve a statistical power of 95% for detecting an effect size of Cohen’s dz = 0.86 (non-centrality parameter δ = 3.84, critical *t* = 2.09, actual power = 0.9535) (26).

To account for potential attrition, defined as patients requiring early extubation, transition to noninvasive ventilation, change in hemodynamics, withdrawal of consent, inadequate signal quality, or technical recording issues, the target enrollment was increased by 20%, resulting in a planned final sample size of 25 patients. Under the same effect size and α, this sample retains approximately 95% power, and would exceed this if all 25 participants complete both intervention periods. This sample size ensures sufficient sensitivity to detect clinically meaningful differences in synchronization performance between the two study modes.

### 2.10 Statistical analyses

The normality of data distribution was assessed using the Shapiro–Wilk test in combination with evaluations of skewness and kurtosis. Continuous variables were expressed as mean and standard deviation (SD) for normally distributed data, or as median and interquartile range (IQR) for non-normally distributed data. Categorical variables were reported as absolute numbers and percentages.

Comparisons between synchronization modes (IS+ and conventional control) were performed using paired statistical tests. For normally distributed variables, the paired-samples *t*-test was applied, whereas the Wilcoxon signed-rank test was used for non-normally distributed data. The primary outcome, the Asynchrony Index (AI), and secondary outcomes including the Major Asynchrony Index, Minor Asynchrony Index, Comfort-B (Behavioral) Scale, percentage of leak, mean SpO_2_, and mean EtCO_2_, were analyzed accordingly.

All tests were two-tailed, and a *p*-value of < 0.05 was considered statistically significant. Data processing, signal synchronization, and event detection were performed using MATLAB (version 2023b; The MathWorks, Inc., Natick, MA, United States). Statistical analyses were conducted with XLSTAT (version 2022; Addinsoft, Paris, France), and visualizations were generated using GraphPad Prism (version 10; GraphPad Software, San Diego, CA, United States) and JASP (version 2023; JASP Team, Amsterdam, Netherlands).

## 3 Results

### 3.1 Patient enrollment and exclusions

**FIGURE 1:**
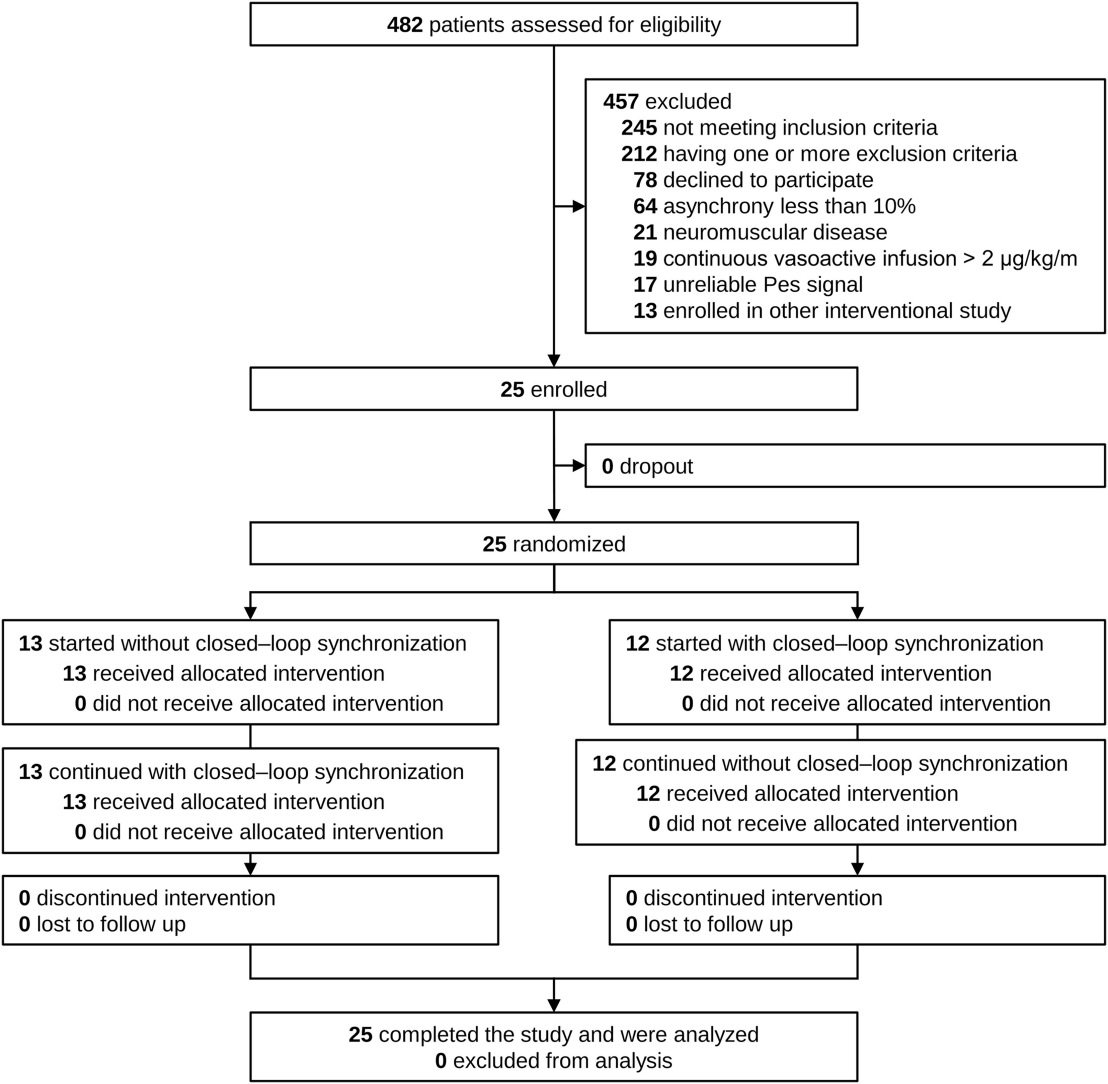
Trial consort profile.

Between March 2024 and May 2025, a total of 482 patients were assessed for eligibility. Of these, 457 patients were excluded — 245 for not meeting inclusion criteria, 212 for meeting one or more exclusion criteria, 78 declined to participate, 64 had an asynchrony index below 10%, 21 had neuromuscular disease, 19 were receiving continuous vasoactive infusions exceeding 2 μg/kg/min, 17 had unreliable esophageal pressure signals, and 13 were enrolled in another interventional study. Ultimately, 25 patients were enrolled and randomized, with 13 initially assigned to the conventional synchronization arm and 12 to the closed-loop synchronization arm. There were no dropouts or losses to follow-up, and all participants completed both intervention phases. Therefore, data from 25 patients were included in the final analysis (Figure 1) (27).

### 3.2 Baseline characteristics

Table 1 summarizes baseline characteristics. Patients were predominantly infants (median age 11 months, IBW 11 kg), with typical ventilator settings (PEEP 5 cmH2O, PS 8.5 cmH2O). The baseline asynchrony index was 16.5% (major 6.4%, minor 10%). Most admissions were respiratory (pneumonia/bronchiolitis, 44%), and lung physiology was mainly mixed or restrictive (72%).

**TABLE 1:** Baseline characteristics of the study cohort.

Variables	Median (IQR 25–75) or mean (SD) or *n* (%)
Gender ratio (%f/%m)	44/56
Age (months)	11 (13–55.5)
IBW (kg)	11 (5–21.5)
PIM3 (%)	4.4 (0.8–19)
PELOD (%)	4 (1–13)
MV duration (days)	5 (3–6.5)
PEEP (cmH_2_O)	5 (5–8)
PS (cmH_2_O)	8.5 (6–5–10.5)
RR (breath/minute)	28 (25–37)
FiO_2_ (%)	30 (23–37.5)
Trigger (L/min)	0.75 (0.5–1)
P-ramp (ms)	50 (37.5–87.5)
ETS (%)	30 (25–40)
AI (%)	
Total	16.5 (13.9–19.3)
Major	6.4 (4.7–8.2)
Minor	10 (8–13)
Admission diagnosis	
Respiratory	11 (44)
A. pneumonia	
A. bronchiolitis	
Sepsis	6 (24)
Neurologic	3 (12)
SE	
Meningoencephalitis	
Renal/metabolic	3 (12)
RTA	
DKA	
Cardiovascular	2 (8)
VSD	
ASD	
Lung physiology	
Mixed	11 (44)
Restrictive	7 (28)
Obstructive	4 (16)
Normal	3 (12)

Data are expressed as median (interquartile range, IQR) or as mean (standard deviation, SD) or number and percentage. IBW, ideal body weight; PIM3, pediatric index of mortality 3, probability of death; PELOD, pediatric logistic organ dysfunction, probability of death; MV, mechanical ventilation; PEEP, positive end expiratory pressure; PS, pressure support; RR, respiratory rate; FiO_2_, fraction of inspired oxygen; P-ramp, pressure rise time; ETS, expiratory trigger sensitivity; AI, asynchrony index; A. pneumonia, acute pneumonia; A. bronchiolitis, acute bronchiolitis; SE, status epilepticus; RTA, renal tubular acidosis; DKA, diabetic ketoacidosis; VSD, ventricular septal defect; ASD, atrial septal defect.

### 3.3 Primary and secondary outcomes

**FIGURE 2:**
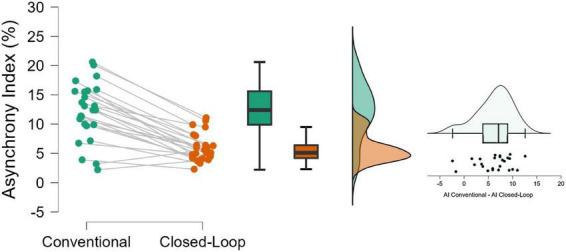
Primary outcome, asynchrony index: effect of closed-loop (CL) synchronization compared with conventional physician-tailored synchronization on overall patient–ventilator asynchrony. The left panel shows paired circles connected with lines, each representing an individual patient, illustrating consistently lower Asynchrony Index (AI) values during CL than during conventional synchronization. The middle boxplots (median, IQR, whiskers) depict the overall reduction in AI with IS+. The right paired-difference plot (Conventional–CL) shows mostly positive values, confirming that AI was lower under CL across the cohort.

When closed-loop synchronization (IS+) was enabled, the Asynchrony Index (AI) was significantly lower compared with conventional physician-tailored synchronization (median 5.1% [IQR 4.2–6.4] vs. 12.4% [IQR 9.9–15.6]; median difference -6.6% [95% CI −8.0 to −4.9]; *P* < 0.001) (Figure 2 and Table 2).

**TABLE 2:** Primary and secondary outcomes.

Variable	Closed-loop	Conventional	Median difference (95%CI)	*P*-value
Primary outcome				
AI (%)	5.1 (4.2 to 6.4)	12.4 (9.9 to 15.6)	–6.6 (–8 to –4.9)	**>0.001**
Secondary outcomes				
Major AI (%)	2.6 (2.1 to 3.3)	4.8 (3.8 to 6.0)	–2.2 (–3.1 to –1.4)	**>0.001**
DB	0.1 (0 to 0.2)	0.8 (0.3 to 1.7)	–1 (–1.5 to –0.6)	**>0.001**
AT	0.4 (0.2 to 0.8)	1.2 (0.7 to 2)	–0.9 (–1.3 to –0.6)	**>0.001**
IE	1.8 (1.4 to 2.7)	1.8 (1.2 to 3.1)	0.001 (–0.5 to –0.3)	0.97
Minor AI (%)	2.6 (2.3 to 3.5)	7.7 (4.9 to 9.4)	–4.3 (–5.4 to –2.7)	**>0.001**
TD	0.6 (0.3 to 0.8)	3.5 (1.8 to 4.3)	–2.5 (–3.5 to –1.7)	**>0.001**
EC	0.2 (0.1 to 0.3)	2.8 (1.9 to 3.9)	–2.7 (–3.7 to –1.9)	**0.001**
LC	1.8 (1 to 2.7)	0.7 (0.2 to 0.9)	1.2 (0.8 to 1.9)	**<0.001**
Comfort B	13 (13 to 14.5)	14 (13 to 15)	–1 (–1.0 to –0.001)	**0.02**
EtCO_2_ (mmHg)	42 (39 to 44)	43 (40 to 45)	–1.5 (–2 to –1)	**>0.001**
SpO_2_ (%)	96 (94 to 97)	96 (94 to 97)	1 (0.001 to 1.0)	0.07
Leak (%)	6 (4 to 7)	6 (4 to 7)	0.001 (–0.5 to 1)	0.48

Data are expressed as median (interquartile range, IQR) or as mean (standard deviation, SD). Wilcoxon or student’s *t*-test were performed depending on each variable distribution according to the Shapiro–Wilk test. 95% CI, 95% confidence interval; AI, asynchrony index; DB, double breath; AT, auto-trigger; IE, ineffective effort; TD: trigger delay; EC, early cycle; LC, late cycle; EtCO_2_, end-tidal carbon dioxide; SpO_2_, peripheral oxygen saturation. Bold *p* values were statistically significant.

**FIGURE 3:**
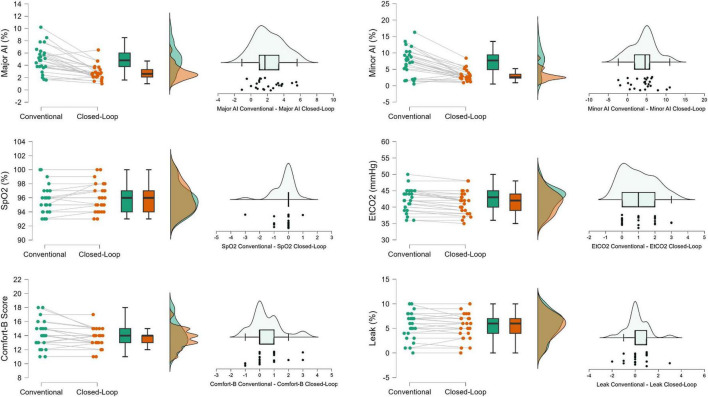
Effect of closed-loop (CL) synchronization compared with conventional physician-tailored synchronization on secondary outcomes. Each pair of circles connected with lines represents an individual patient, illustrating consistently lower Major and Minor Asynchrony Index (AI) values during CL. EtCO_2_ levels were also lower, reflecting improved ventilatory efficiency. Comfort-B scores showed a modest but significant improvement, while SpO_2_ and Leak remained unchanged between the two modes. Boxplots (median, IQR, whiskers) demonstrate these overall trends across the cohort, and paired-difference plots on the right confirm the consistent direction of change favoring CL in most patients.

IntelliSync+also markedly reduced major and minor asynchrony indices (-2.2% [95% CI −3.1 to −1.4] and −4.3% [95% CI −5.4 to −2.7], respectively; *P* < 0.001 for both) (Figure 3 and Table 2).

Among asynchrony subtypes, the frequencies of double triggering (DB), auto-triggering (AT), trigger delay (TD), and early cycling (EC) were all significantly lower during IS+ (*P* ≤ 0.001), while ineffective efforts (IE) remained unchanged (*P* = 0.97). In contrast, late cycling (LC) occurred slightly more frequently under IS+ (*P* < 0.001) (Table 2).

Closed-loop synchronization maintained patient comfort and leak stability (Comfort-B 14 [IQR 13–14] vs. 14 [IQR 13–15]; *P* = 0.02; Leak 6% [IQR 4–7] vs. 6% [IQR 4–7]; *P* = 0.48), and improved ventilatory efficiency as indicated by a lower end-tidal CO_2_ (42 mmHg [IQR 39–44] vs. 43 mmHg [IQR 40–45]; *P* < 0.001) without significant change in oxygen saturation (*P* = 0.07) (Figure 3 and Table 2).

## 4 Discussion

This multicenter, prospective, randomized controlled crossover trial demonstrates that the implementation of the IS+ closed-loop synchronization algorithm significantly reduces PVA compared with conventional physician-tailored synchronization settings in pediatric patients receiving invasive mechanical ventilation. The primary finding of this study was a marked reduction in the overall AI under IS+, indicating enhanced synchronization between the ventilator and the patient’s spontaneous respiratory efforts.

The reduction in total AI was driven by improvements in both major and minor asynchrony indices. Among major asynchronies, IS+ significantly decreased the incidence of AT and DB, while among minor asynchronies, TD and EC were markedly reduced. Only LC occurred slightly more frequently under IS+, likely due to heightened sensitivity in detecting patient effort rather than a mechanical limitation.

At baseline, the overall asynchrony burden in our cohort was lower than that reported by Mortamet et al. in invasively ventilated pediatric patients (28). While trigger delay, ineffective efforts, and cycling errors were the predominant asynchrony types in our study, their reported frequencies were consistently lower than those previously described. In contrast, Mortamet et al. observed higher rates of trigger delay and late cycling, and Blokpoel et al. reported that more than one third of ventilated breaths were asynchronous, with ineffective efforts and late cycling accounting for the majority of events (3). Compared with these findings, the distribution pattern of asynchrony types in our cohort differed, with trigger- and cycling-related events being more prominent than ineffective efforts. This variability likely reflects differences in patient characteristics, ventilator performance, and the specific definitions and differences in measurement methodology used across studies, as their analysis included the entire duration of mechanical ventilation, whereas our baseline assessment was limited to a stable measurement period prior to the randomized interventions. Despite these differences, nearly one in six breaths in our cohort remained asynchronous, underscoring that patient–ventilator asynchrony persists as a clinically relevant issue even with modern ventilators and optimized conventional settings.

Following these baseline measurements, patient–ventilator synchrony was optimized by the attending physicians according to clinical judgment, and after a 30-min run-in period, data were recorded for 1 h under conventional physician-tailored synchronization. During these conventional periods, the overall AI was 12.4% (IQR 9.9–15.6), consisting of a major AI of 4.8% (IQR 3.8–6.0) and a minor AI of 7.7% (IQR 4.9–9.4). Among major asynchronies, the most frequent event was IE with a median of 1.8% (IQR 1.2–3.1), while among minor asynchronies, TD was the most common, occurring at a median of 3.5% (IQR 1.8–4.3). Compared with the baseline period, both total AI and the frequencies of the most common asynchrony types were modestly reduced, reflecting the short-term benefits of individualized ventilator adjustments by the clinical team. However, despite physician optimization, asynchrony events persisted, indicating that manual adjustments alone were insufficient to achieve full synchrony even under controlled conditions.

When the IS+ closed-loop synchronization was activated, the overall AI decreased significantly from 12.4% (IQR 9.9–15.6) during conventional physician-tailored synchronization to 5.1% (IQR 4.2–6.4) (*P* < 0.001). This reduction was accompanied by a decline in both major and minor asynchrony indices, which fell from 4.8% (IQR 3.8–6.0) and 7.7% (IQR 4.9–9.4) to 2.6% (IQR 2.1–3.3) and 2.6% (IQR 2.3–3.5), respectively (both *P* < 0.001). Among the major asynchronies, IE remained the most frequent event but showed no significant change between modes (1.8% [IQR 1.4–2.7] vs. 1.8% [IQR 1.2–3.1]; *P* = 0.97). In contrast, both AT and DB were significantly reduced under IS+ (0.4% [IQR 0.2–0.8] vs. 1.2% [IQR 0.7–2.0] and 0.1% [IQR 0–0.2] vs. 0.8% [IQR 0.3–1.7], respectively; both *P* < 0.001), accounting for the overall decrease in major AI.

Similarly, within minor asynchronies, TD and EC were markedly reduced under IS+ (0.6% [IQR 0.3–0.8] vs. 3.5% [IQR 1.8–4.3] and 0.2% [IQR 0.1–0.3] vs. 2.8% [IQR 1.9–3.9]; both *P* ≤ 0.001), whereas LC increased slightly (1.8% [IQR 1.0–2.7] vs. 0.7% [IQR 0.2–0.9]; *P* < 0.001). Despite this rise in LC, the overall minor AI still decreased significantly, confirming a net improvement in synchrony across both trigger- and cycling-related events.

In this comparison between the closed-loop (IS+) and conventional physician-tailored modes, leak percentage and SpO_2_ remained unchanged (*P* > 0.05). However, patients demonstrated a statistically significant improvement in comfort (*P* = 0.02) and ventilatory efficiency as indicated by reduced EtCO_2_ levels (*P* < 0.001), suggesting enhanced synchronization and more effective ventilation under the closed-loop system.

de la Oliva et al. demonstrated that reducing asynchronies can have a positive impact on patient comfort. In their study, the asynchrony index (AI) was significantly lower during neurally adjusted ventilation, mainly due to decreases in auto-triggered and non-triggered breaths, while double triggering showed no significant change. Although the sedative dose remained constant, the Comfort scale significantly improved during neurally adjusted ventilation compared with optimized pressure support ventilation (16). This observation was later supported by a similar study (29). In line with these findings, our study also demonstrated that the IS+ closed-loop synchronization reduced asynchronies and was associated with a modest but significant improvement in Comfort-B scores, further supporting the hypothesis that enhanced synchrony directly improves patient comfort during invasive mechanical ventilation.

Together, these findings highlight the capacity of IS+ to dynamically adjust inspiratory and expiratory cycling in real time based solely on conventional airway pressure and flow waveform analysis, optimizing synchronization without compromising comfort, safety, or gas exchange.

Previous pediatric studies have reported high rates of patient–ventilator asynchrony under conventional modes, emphasizing the need for a better solution in achieving precise synchrony (3, 12, 28). Unlike those observational analyses, the present trial provides the first prospective evidence that a closed-loop synchronization algorithm that operates solely through pressure and flow waveform analysis can effectively reduce asynchrony in invasively ventilated children.

In line with our previous research investigating other closed-loop ventilation and oxygenation systems, the present findings support the broader principle that automated control enhances the percentage of time spent within optimal physiological ranges. Prior studies on closed-loop ventilation strategies have demonstrated that these systems are more effective than conventional methods in reducing driving pressure and optimizing oxygenation efficiency, thereby ensuring safer and more precise ventilatory management (30–33). Similarly, IS+ achieves improved patient–ventilator interaction by continuously analyzing airway pressure and flow signals to anticipate and match patient effort.

Taken together, these findings reinforce the concept that closed-loop synchronization can enhance the precision of ventilatory support, reduce variability, and minimize the need for clinician intervention. The CHESTSIPP study thus extends the evidence base for closed-loop systems in pediatric intensive care by demonstrating, for the first time, that closed-loop synchronization can significantly improve interaction between patient and ventilator during invasive mechanical ventilation. Recent evidence has emphasized the potential of automated decision-support (CDS) systems in optimizing ventilatory management. Khemani et al. demonstrated that a lung- and diaphragm-protective ventilation strategy guided by a CDS tool during the acute phase of mechanical ventilation significantly shortened the duration of weaning compared with usual care. Moreover, in their study, the success of spontaneous breathing trials (SBTs) was assessed using the Pressure–Rate Product (PRP), highlighting the importance of integrating physiologic monitoring tools into ventilator management (34, 35).

In another investigation, patients who maintained better synchrony with the ventilator exhibited lower pressure–time product (PTP) values, indicating reduced inspiratory effort and improved respiratory muscle efficiency compared with asynchronous patients (36).

Considering these findings, the results of the current trial suggest that closed-loop synchronization systems such as IS+, by effectively reducing asynchronies, may help optimize parameters associated with lung–diaphragm protection, reduce respiratory workload, and potentially facilitate earlier and more successful extubation. However, there is currently no direct evidence confirming that improvements in synchrony translate into shorter weaning duration or higher extubation success rates, particularly in pediatric patients. Future studies should therefore aim to determine whether these short-term physiological improvements provided by closed-loop synchronization ultimately result in better clinical outcomes, including reduced duration of ventilation and improved extubation success.

This study has several limitations. First, it was conducted over a relatively short observation period and included only hemodynamically stable patients, which may limit the generalizability of the findings to more critically ill populations. Second, although the crossover design minimized interindividual variability, it also confined the evaluation to short-term physiological effects rather than long-term clinical outcomes. There is currently no direct evidence confirming that improvements in patient–ventilator synchrony translate into shorter weaning duration or higher extubation success rates, particularly in pediatric patients. Third, the study focused exclusively on patients with preserved spontaneous breathing activity; therefore, its results cannot be extrapolated to those requiring controlled ventilation or high levels of sedation.

## 5 Conclusion

In this multicenter randomized crossover trial, the IS+ closed-loop synchronization algorithm significantly reduced patient–ventilator asynchrony compared with conventional physician-tailored synchronization in pediatric patients undergoing invasive mechanical ventilation. The reduction in both major and minor asynchronies was achieved without compromising comfort, gas exchange, or safety, and was associated with improved ventilatory efficiency. These findings suggest that closed-loop synchronization represents a promising step toward automated, physiology-based ventilatory management in children. Larger and longer studies are warranted to determine whether these short-term physiological improvements translate into tangible clinical benefits such as shorter weaning duration and improved extubation success.

## Data Availability

The raw data supporting the conclusions of this article will be made available by the authors, without undue reservation.

## Supplementary material

Supplementary Figure 1 Study protocol. A 30-min baseline under conventional ventilation preceded two randomized 90-min intervention phases; each included a 30-min adaptation (run-in) and a 60-min measurement period. A 30-min washout between interventions also served as the subsequent run-in, ensuring physiological re-stabilization before the next phase.
