# Supplementary figures and images for: Closed-loop synchronization versus conventional synchronization in spontaneously breathing pediatric patients (CHESTSIPP) – a randomized controlled cross-over study
Source: Front Med (Lausanne). 2026 Mar 25;13:1745939. doi: 10.3389/fmed.2026.1745939 (PMC13057382; doi:10.3389/fmed.2026.1745939)

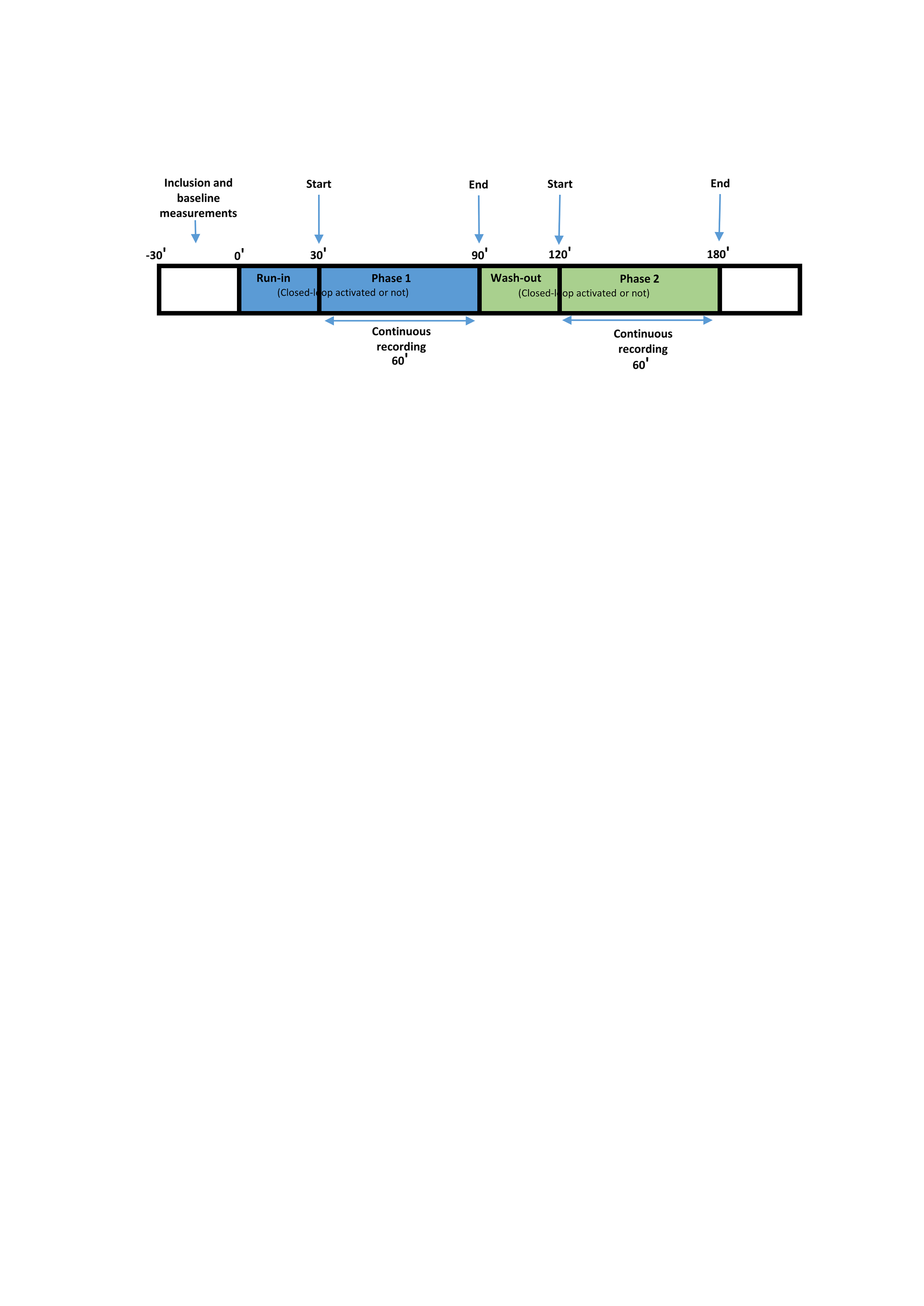

Supplement: Supplementary Figure 1 — Study protocol. A 30-min baseline under conventional ventilation preceded two randomized 90-min intervention phases; each included a 30-min adaptation (run-in) and a 60-min measurement period. A 30-min washout between interventions also served as the subsequent run-in, ensuring physiological re-stabilization before the next phase. [file Image_1.tiff]
